# Evidence-Based Network Approach to Recommending Targeted Cancer Therapies

**DOI:** 10.1200/CCI.19.00097

**Published:** 2020-01-28

**Authors:** Jayaram Kancherla, Shruti Rao, Krithika Bhuvaneshwar, Rebecca B. Riggins, Robert A. Beckman, Subha Madhavan, Héctor Corrada Bravo, Simina M. Boca

**Affiliations:** ^1^ University of Maryland, College Park, MD; ^2^ Georgetown University, Washington, DC

## Abstract

**PURPOSE:**

In this work, we introduce CDGnet (Cancer-Drug-Gene Network), an evidence-based network approach for recommending targeted cancer therapies. CDGnet represents a user-friendly informatics tool that expands the range of targeted therapy options for patients with cancer who undergo molecular profiling by including the biologic context via pathway information.

**METHODS:**

CDGnet considers biologic pathway information specifically by looking at targets or biomarkers downstream of oncogenes and is personalized for individual patients via user-inputted molecular alterations and cancer type. It integrates a number of different sources of knowledge: patient-specific inputs (molecular alterations and cancer type), US Food and Drug Administration–approved therapies and biomarkers (curated from DailyMed), pathways for specific cancer types (from Kyoto Encyclopedia of Genes and Genomes [KEGG]), gene-drug connections (from DrugBank), and oncogene information (from KEGG). We consider 4 different evidence-based categories for therapy recommendations. Our tool is delivered via an R/Shiny Web application. For the 2 categories that use pathway information, we include an interactive Sankey visualization built on top of d3.js that also provides links to PubChem.

**RESULTS:**

We present a scenario for a patient who has estrogen receptor (ER)–positive breast cancer with *FGFR1* amplification. Although many therapies exist for patients with ER-positive breast cancer, *FGFR1* amplifications may confer resistance to such treatments. CDGnet provides therapy recommendations, including PIK3CA, MAPK, and RAF inhibitors, by considering targets or biomarkers downstream of FGFR1.

**CONCLUSION:**

CDGnet provides results in a number of easily accessible and usable forms, separating targeted cancer therapies into categories in an evidence-based manner that incorporates biologic pathway information.

## INTRODUCTION

In today’s era of cancer precision medicine, therapeutic interventions are often tailored to an individual’s tumor molecular profile, in addition to traditional considerations, including age, sex, cancer stage, and medical and treatment histories. The term molecular profiling is often used to refer to a test that considers ≥1 biomarker. These biomarkers may be either genetic characteristics or mRNA or protein expression values. Genetic characteristics include point mutations, insertions, deletions, duplications, gene fusions, and rearrangements. They may be either germ line (inherited and present in normal tissue) or somatic (present in cancer cells but not normal tissue). Expression values refer to the expression of mRNA or protein in tumors, either in comparison with other tumors or adjacent normal tissue. Typically, tumor molecular profiling is used when a patient has few or no standard treatment options left. However, for some tumor types, it is now routine to check for specific molecular features to decide on targeted treatment plans. For example, *KRAS* wild-type colorectal cancer is generally treated with epidermal growth factor receptor (EGFR) inhibitors,^1^ estrogen receptor (ER)–positive breast cancer with aromatase inhibitors or antiestrogens such as tamoxifen or fulvestrant, and human epidermal growth factor receptor 2–positive breast cancer with monoclonal antibodies trastuzumab and pertuzumab, tyrosine kinase inhibitors such as neratinib, or antibody-toxin conjugates such as trastuzumab-DM1.^2^ In many cases, if there is no US Food and Drug Administration (FDA)–approved targeted therapy for a specific tumor type, clinicians may recommend either an off-label therapy that is prescribed for the patient’s alteration in another tumor type or enrollment in a precision medicine clinical trial (eg, basket, umbrella, or targeted therapy trial).

**CONTEXT**

**Key Objective**
With the increasing use of tumor molecular profiling, it is imperative to develop approaches that consider the biologic context to better prioritize targeted therapies for patients with cancer.
**Knowledge Generated**
We introduce CDGnet (Cancer-Drug-Gene Network), a tool that integrates biologic pathway information with alterations detected from tumor molecular profiling to expand the possible range of targeted therapies and prioritize them into evidence-based categories. We demonstrate how CDGnet can be applied to a scenario where a patient with breast cancer has overexpression of both *ESR1* and *FGFR1*, with the output representing the recommended therapies, the clinical context in which they are approved, and the links between the patient’s tumor molecular profile and the recommendations.
**Relevance**
We consider this tool to be especially valuable to clinical and translational researchers who may be interested in understanding the best course of treatment for patients with a particular tumor molecular profile.

**FIG 1.**
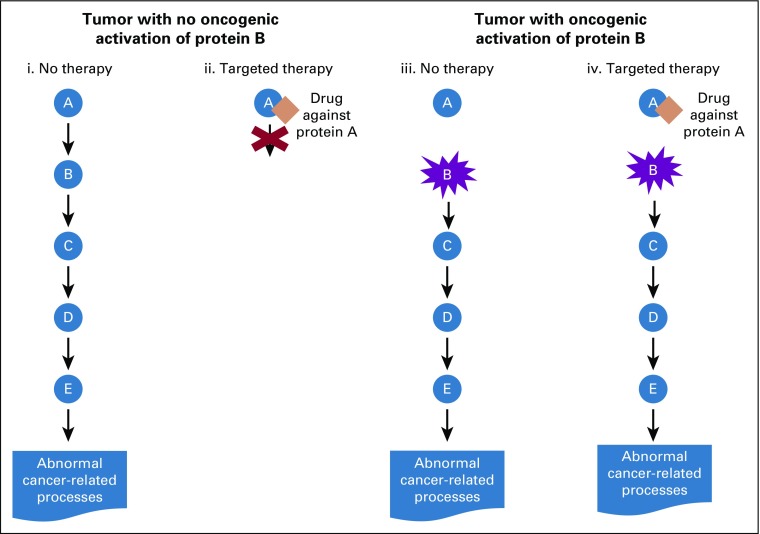
Simplified diagram showing the reasoning behind looking at downstream targets of activated oncogenes. A simple pathway is shown that consists of 5 proteins, A, B, C, D, and E, with A activating B, B activating C, and so on, with the final activation of E leading to various abnormal cancer-related processes. (i, ii) Scenario where a tumor has no oncogenic activation of protein B. (iii, iv) Scenario where protein B has gained an oncogenic mutation that renders it constitutively active. If there is no oncogenic activation of protein B, then targeting protein A, as in (ii), may be effective in stopping cancer growth. However, if there is oncogenic activation of protein B, this means that, in particular, it is not necessary for protein A to activate protein B, so that targeting protein A is not effective for turning off the pathway.

To make such decisions about off-label therapy recommendations, clinicians have to sift through vast amounts of literature and clinical databases to determine the clinical utility of variants identified through molecular profiling to decide on the appropriate treatment option for their patients. The same is true for clinical translational scientists considering relevant therapeutic approaches to evaluate in model systems or humans, using either single agents or combinations. In this setting, the number of possible molecular profiles that may be relevant and the number of experimental agents create a combinatorial explosion of research possibilities among which prioritization is needed. Several efforts are ongoing to capture, standardize, and share clinically relevant variants identified through molecular diagnostic tests among several public, academic, and private institutions,^3-5^ although challenges remain in synthesizing evidence in a manner that is both systematic and timely.^6,7^ Our goal in this work is to expand the range of options for targeted therapies for patients with cancer who undergo molecular profiling by developing CDGnet (Cancer-Drug-Gene Network), a user-friendly, evidence-based approach that accounts for downstream effects within pathways in cancer and is personalized for the individual patient. Our tool, which uses the Shiny framework with an R backend,^8^ is available online.^9^ We incorporate pathway information specifically by looking at downstream targets of oncogenes, which are genes that are constitutively activated in cancer.^10^ This is illustrated in Figure 1. If an oncogene in a biologic pathway is activated, targeting genes and proteins that are found upstream may no longer be effective, leading to a focus on downstream targets. This includes the scenario of EGFR inhibitors for *KRAS* wild-type colorectal tumors. The EGFR protein triggers a signaling cascade in cancer, which may be blocked by anti-EGFR drugs; however, this is only effective if *KRAS*, which is downstream of EGFR, is not mutated. Otherwise, certain *KRAS* mutations can lead to a lack of effectiveness of therapies that block EGFR. As a result, patients with colorectal cancer are typically tested for *KRAS* mutations, and EGFR inhibitors are only prescribed to individuals without specific *KRAS* mutations in codons 12 and 13. A comprehensive characterization of untreated colorectal tumors estimated that 43% of nonhypermutated tumors had *KRAS* mutations, and these mutations were generally oncogenic activating mutations,^11^ which means that a large percentage of patients with colorectal cancer are left with few therapeutic options. Our framework and tool are seeking to remedy this issue.

## METHODS

### Overview of Methods for Generating Patient-Specific Networks

**FIG 2.**
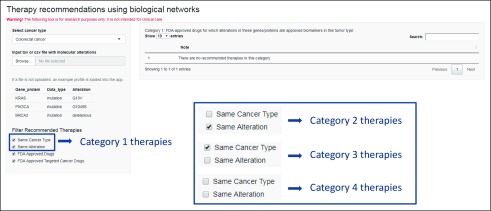
Part of the landing page, which shows how users can select the cancer type and either input a tab-separated or comma-separated file or use the example data. The inset shows how under “Filter Recommended Therapies,” combinations of the first 2 checkboxes lead to the 4 different categories of therapy recommendations described in the text. Removing 1 or both of the last 2 checkboxes expands the range of therapies in categories 3 and 4 beyond US Food and Drug Administration (FDA)–approved drugs and FDA-approved targeted cancer drugs, respectively.

The user inputs into CDGnet are the specific alterations found in a patient’s tumor and the patient’s cancer type. Part of the landing page is shown in Figure 2. These data are then integrated with biologic networks relevant to the cancer type (from the Kyoto Encyclopedia of Genes and Genomes [KEGG] database^12^), FDA-approved targeted cancer therapies and indications (curated from DailyMed therapy labels^13^), additional gene-drug connections in the form of drug targets (from the DrugBank database^14^), information on whether a gene is an oncogene (from KEGG). Users may consider different data sources by using the CDGnet code^15^ directly, for example, by considering the predicted oncogenes from a recent comprehensive characterization of The Cancer Genome Atlas (TCGA) projects.^16^ Currently, the biologic networks we consider are the cancer-specific pathways in KEGG, and therefore, for now, we are also restricting the cancer types to those that have KEGG pathways. We have developed 4 different therapy categories that can be prioritized for patients, given their specific tumor alterations, ordered from “most evidence that therapy works” to “least evidence that therapy works.” (1) FDA-approved drugs for which the input genes/proteins are biomarkers for their tumor type; (2) FDA-approved drugs for which the input genes/proteins are biomarkers in other tumor types; (3) drugs that have as targets the input genes/proteins or as biomarkers/targets other genes/proteins that are downstream of the input oncogenes when considering the pathway corresponding to this tumor type; and (4) drugs that have as biomarkers/targets other genes/proteins that are downstream of input oncogenes when considering the pathways corresponding to other tumor types.

In categories 3 and 4, users have the option to consider only FDA-approved targeted cancer therapies, all FDA-approved therapies, or all drugs in DrugBank; this allows clinical researchers to consider increasing numbers of therapies only as needed, as opposed to being overwhelmed with a huge number of therapies from the start. We also note the difference between categories 3 and 4; category 3 considers the biologic pathway corresponding to the individual’s cancer type, whereas category 4 considers the pathways corresponding to other cancer types. Given that pathways represent a simplification of a more complicated reality and each tumor is unique, we found it necessary to allow for possible connections between genes and proteins that may be curated in cancer types different from that with which a patient presents, although in our experience, it is generally sufficient to stop at category 3 therapies.

**FIG 3.**
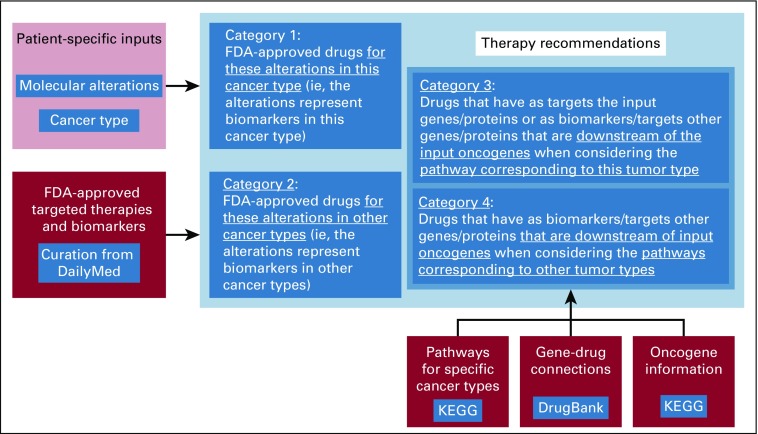
General approach for targeted therapy recommendations, including specific data sources. FDA, US Food and Drug Administration; KEGG, Kyoto Encyclopedia of Genes and Genomes.

We differentiate between targets and biomarkers because in many cases, as a result of complicated biologic interactions, the target of a therapy may be different from the biomarker used to specify the indication, such as in the case of EGFR inhibitors being administered for *KRAS* wild-type colorectal tumors or CDK4/6 inhibitors being administered for ER-positive breast tumors. The general approach is presented in Figure 3. The options used on the landing page to obtain the different therapy categories are shown in Figure 2. We also provide documentation for the tool, including a step-by-step analysis for the built-in patient use case scenario.^17^

Appendix Table A1 lists FDA-approved targeted cancer therapies and indications that were obtained by considering the targeted therapies listed by the National Cancer Institute^18^ and looking up the corresponding labels via DailyMed.^13^ In particular, the indications and usage portion of the label was used to obtain the specific cancer type and biomarker information, which is listed in the “Gene/Protein,” “Data Type,” and “Alteration” columns; in the case of multiple biomarkers, these are listed in separate rows of the table. In cases where the biomarker indication is unclear, the lists of FDA companion diagnostic tests were also consulted.^19,20^ Note that although some targeted therapies have specific biomarker indications, many do not. For example, ibrutinib is a targeted therapy, administered for a number of subtypes of leukemia/lymphoma, but not for a specific biomarker indication. If there is no biomarker indication, this is noted as an asterisk in the table in the “Gene/Protein” column. The therapies are then cross-referenced with DrugBank to obtain the targets for both the therapies with biomarker indications and those without indications. The biomarkers and targets obtained in these ways are checked against downstream targets from KEGG cancer-specific pathways, which were downloaded and parsed and had identifiers converted using the KEGGREST,^21^ KEGGgraph,^22^ and org.Hs.eg.db^23^ Bioconductor packages, respectively, and against the information input by the user, with the gene/protein names being normalized via the rDGIdb package, which is a wrapper for the Drug Gene Interaction Database.^24,25^

To obtain the list of FDA-approved drugs, we used the data files from the official Drugs@FDA resource.^26^ Drugs@FDA contains several tab-separated value files that include information on the submission, review, and approval process for various drugs. We use the products (list of all drugs) and submission (review process for all drugs) files to filter for drugs that are approved or tentatively approved. The Drugs@FDA resource contains a list of all drugs approved since 1939, some of which may have been discontinued. As a result, we use the marketingstatus file to remove any discontinued products from the list. The R scripts to parse and filter the Drugs@FDA data files are available in our GitHub repository.

### Shiny App and Visualization

**FIG 4.**
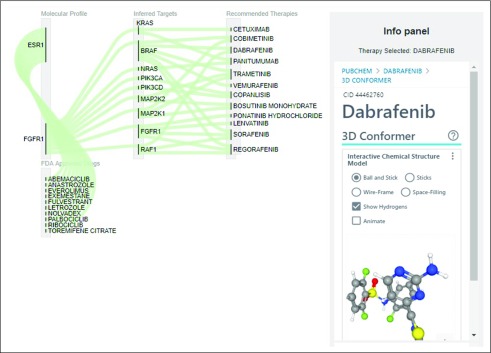
Sankey flow diagram focusing on the flow of evidence between drug-gene and gene connections for a putative patient with estrogen receptor–positive breast cancer and FGFR1 overexpression, showing category 3 recommendations, namely, targets downstream of FGFR1. Therapies can be clicked to obtain a panel with PubChem information.

**FIG 5.**
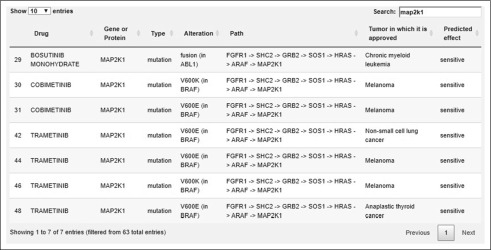
Part of the sortable, searchable table for therapies in category 3 for a putative patient with estrogen receptor–positive breast cancer and FGFR1 overexpression, showing the subset of therapies that target MAP2K1.

For each of the 4 categories detailed, a sortable and searchable table of therapies is output with the FDA-approved indications; for categories 3 and 4, network visualizations are also shown. The table also provides the tumor type in which a particular therapy is approved. Figure 4 shows a Sankey flow diagram representation that focuses on the flow of evidence between drug-gene and gene-gene connections, enabling an intuitive visualization from the molecular profile to the inferred targets and recommended therapies. Figure 5 shows a portion of the sortable and searchable corresponding table. The path column represents the pathway between the altered gene/protein and the gene/protein that is a biomarker or target; the alteration column represents the biomarker for an FDA-approved indication, if this exists, in which case the tumor for which it is approved is also listed; the predicted effect column has the value “sensitive”, if the alteration column is not empty, or “target”, if the drug targets the protein according to the DrugBank data.

**FIG 6.**
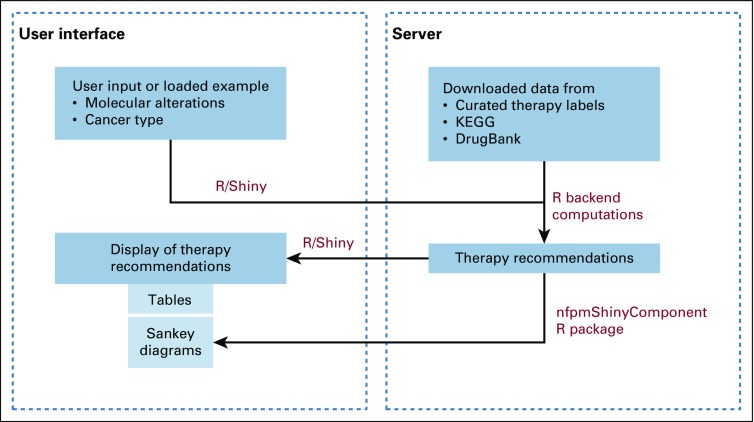
Architecture diagram for our system. KEGG, Kyoto Encyclopedia of Genes and Genomes.

An architecture diagram for our system is shown in Figure 6. We use Shiny, an R package/framework for creating interactive and standalone Web applications directly from R.^8^ Shiny applications can run on a Web page or be embedded in RMarkdown documents to build interactive dashboards. They use the same technology that powers Web applications (ie, HTML and JavaScript) and allow users to create intuitive and interactive user interfaces and prototypes with an R computational backend.

To support interactive Sankey charts within Shiny applications, we developed a Shiny Web component for visualizing Sankey flow diagrams, available to download as an R package.^27^ Web components are custom HTML elements that are natively extensible and reusable and can be integrated into any framework that supports HTML. The Sankey visualization uses a custom 3-column layout to organize nodes in the graph: molecular profile and FDA-approved drugs, inferred targets, and recommended therapies; it intuitively focuses the user on the flow of evidence from input parameters to recommended therapies. The Sankey visualization also contains an information panel that displays evidence related to a pathway connection or information on a drug when a user selects/clicks on an edge or node. Selecting an edge shows the downstream pathway information used for inference. Selecting a recommended therapy displays the structure of the drug and linked publications from PubChem,^28^ using PubChem widgets. The Sankey visualization is built on top of d3.js,^29^ a data visualization library for JavaScript to build highly customizable and interactive visualizations.

## RESULTS

We will now consider the scenario of a patient who has ER-positive breast cancer. ER-positive breast cancer, generally treated with aromatase inhibitors or antiestrogens, employs an array of mechanisms that permit escape from these therapies. These include amplification or upregulation of fibroblast growth factor receptor 1 (*FGFR1*), which is amplified in approximately 13% of ER-positive tumors from TCGA^30-32^ and leads to ligand-independent ER activation.^33^ FGFR activity has also recently been shown to confer resistance to CDK4/6 inhibitors in ER-positive breast cancer.^34^ Pan-FGFR antagonists have been combined with endocrine therapies in prior clinical studies (eg, CTKI258A2210), but the efficacy of this combination has been minimal, even in patients preselected for alterations in the FGFR pathway.^35^ A potential underlying explanation for this lack of benefit is that *FGFR* alterations impinge upon downstream signaling networks shared by many other receptor tyrosine kinases. Figure 4 shows CDGnet recommendations for a patient with breast cancer with overexpression of both *ESR1* (gene encoding ER) and *FGFR1*, when considering only FDA-approved targeted therapies. Therapy recommendations include PIK3CA, MAPK, and RAF inhibitors, which may have utility in this context, along with the standard targeted therapies prescribed for ER-positive breast cancer. Figure 5 shows the subset of the corresponding table that consists of FDA-approved MAP2K1 inhibitors, which are approved for either *ABL1* fusions in chronic myeloid leukemia, or specific *BRAF* mutations in melanoma, non–small-cell lung cancer, and anaplastic thyroid cancer.

## DISCUSSION

We developed the CDGnet tool using an approach that considers biologic pathways and connections among genes, proteins, and drugs to prioritize targeted therapies for patients with cancer. Our approach integrates many disparate sources of knowledge and provides results in an easily accessible and usable format. With our tool, users are able to quickly obtain information on the FDA-approved therapies (category 1) and potential off-label therapies (category 2) associated with a patient’s molecular profile. Our definitions of categories 1 and 2 in CDGnet are in alignment with the tier I and II evidence level classifications recommended by the Association for Molecular Pathology, American College of Medical Genetics and Genomics, ASCO, and College of American Pathologists.^36^ However, CDGnet categories 3 and 4 are unique to our evidence-based network approach and enable users to evaluate additional targeted therapy options based on an individual’s tumor profile. It is important to note that the targeted therapy recommendations in categories 3 and 4 have lower evidence levels and may or may not have proven clinical significance in ongoing clinical trials. However, by examining the downstream targets of candidate biomarkers, clinical researchers can derive key insights into potential biologic pathways that can be targeted by different cancer therapies. On the basis of the level of evidence, the clinical actionability of these pathways can be further tested in a laboratory or clinical trial setting. Additionally, there is a growing field of research related to drug-target interactions and drug repositioning using network-based models,^37-40^ which may in the future be integrated with our tool.

We aim to further enhance the data that drive the CDGnet tool by incorporating relevant information from additional precision oncology efforts, tools, and resources. Users who download or connect to these resources may currently use them in the context of our approach by modifying our code.^15^ Expert-curated precision oncology databases include Clinical Interpretations of Variants in Cancer (CIViC),^5,41^ Cancer Genome Interpreter,^42,43^ OncoKB,^44,45^ Database of Evidence for Precision Oncology (DEPO),^46,47^ and Precision Medicine Knowledge Base (PMKB),^48,49^ and more general resources include ClinVar.^50^ These additional sources may further strengthen the clinical annotations and evidence related to germ line and somatic alterations in our database and provide options between curated drug label information and DrugBank targets. CIViC is an open-access, open-source, community-driven Web resource that allows clinical interpretations of mutations related to cancer. Cancer Genome Interpreter is an online tool that connects genes and drugs along with their effects and publication sources, not in a network format, but in a tabular format. OncoKB is another online precision oncology knowledge base that contains information about the effects and treatment implications of specific cancer gene alterations. DEPO contains druggable variant information such as drug therapies, evidence levels (FDA approved, clinical trials, case reports, and preclinical), and cancer types for intended treatments. PMKB provides information about clinical cancer variants and interpretations. We are also using a simplified model for incorporating pathway information via the consideration of targets that are downstream of oncogenes; there are scenarios we do not capture where upstream targeting can also be useful, for example, in the case of a feedback loop.^51-53^ We will incorporate more complex information in future iterations of our tool. Our tool partly relies on manual curation of information for FDA-approved targeted therapies and thus has challenges similar to those of other tools in this space, including the time- and labor-intensive nature of this process. However, the KEGG and DrugBank components only need to be downloaded and reprocessed through our existing code when updates are desired.

Consortia such as the Clinical Genome Resource Somatic Cancer Working Group^3,54^ and the Global Alliance for Genomics and Health Variant Interpretation for Cancer Consortium^55^ have ongoing efforts to standardize and harmonize the expert-curated data in these different knowledge bases, with the goal of enhancing the interoperability among these databases. We will align the future development of CDGnet with the guidelines and consensus frameworks developed by these consortia. CDGnet can also serve as an informative tool for oncologists, molecular pathologists, and genomic scientists who routinely participate in molecular tumor board discussions.

Tools similar to CDGnet include PreMedKB^56,57^ and the Drug Gene Interaction Network.^58^ PreMedKB is an integrated precision medicine knowledgebase for interpreting relationships among diseases, genes, variants, and drugs. The Drug Gene Interaction Network is a commercial tool offered by Seqome (Tramore, Ireland) that builds drug-gene interaction networks to predict clinical response from multiomics data sets. The advantage of CDGnet over these tools is that our approach allows users to input specific alterations found in a patient’s tumor and cancer type and outputs therapy options ordered based on priority. Such a personalized tool may eventually expand the range of options of targeted therapies for patients with cancer in the clinical setting, a key goal of precision oncology.

We currently consider clinical or translational researchers to be the primary target user group for our tool. For instance, if they are interested in a particular combination of molecular alterations for a specific cancer type and generally find the recommendations to be for drugs prescribed in a different cancer type, they may decide to pursue formal studies of drug repurposing, which is made easier by knowing whether they are considering an FDA-approved targeted drug, FDA-approved drug, or non–FDA-approved drug. Our eventual goal is to allow for the use of this tool by clinicians, especially for the care of patients with advanced-stage disease for whom the immediate FDA-approved therapy choices have been exhausted.

## Figures and Tables

**TABLE A1.**
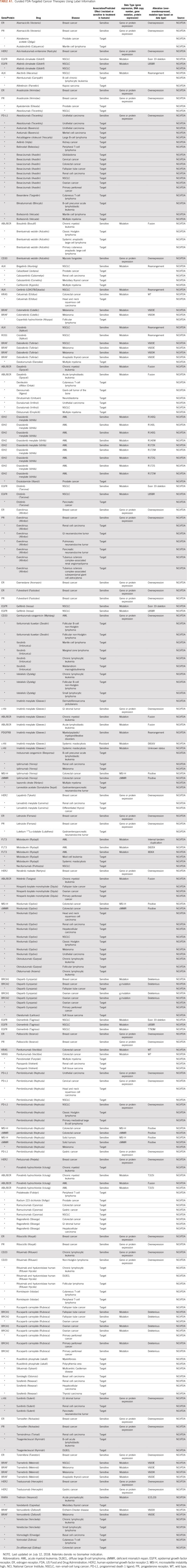
Curated FDA-Targeted Cancer Therapies Using Label Information
